# Impact of Double-J Stent on Patient Outcomes and Healthcare Costs in Saudi Arabia: A Retrospective Cross-Sectional Study From a Tertiary Care Center

**DOI:** 10.7759/cureus.105387

**Published:** 2026-03-17

**Authors:** Khaled Alhussaini, Ahmed Alkhaddam, Haithm Alasim, Mezyed Fahad Alghanim, Diana Hujelan, Khalid Bin Aziz, Fahad Bin Aziz, Hussain Abeery

**Affiliations:** 1 College of Medicine, King Saud bin Abdulaziz University for Health Sciences (KSAU-HS), Riyadh, SAU; 2 Urology, Security Forces Hospital Program, General Directorate of Medical Services, Ministry of Interior, Riyadh, SAU

**Keywords:** double-j stent, healthcare cost, quality of life, saudi arabia, ureteral stent

## Abstract

Background: Double-J (DJ) ureteral stents are commonly used to relieve urinary tract obstruction, yet their placement often leads to pain and other complications that can affect patients’ quality of life (QOL). This study evaluated the impact of DJ stents on patient outcomes and healthcare burden in Saudi Arabia.

Methods: A retrospective cross-sectional study was conducted at Security Forces Hospital, Riyadh, including 135 patients who underwent ureteral stent placement between January 2021 and January 2024. The primary outcome was stent-related quality of life measured using the Ureteral Stent Symptom Questionnaire (USSQ). Data from medical records and telephone-based USSQ interviews were analyzed using descriptive statistics and logistic regression in IBM SPSS Statistics, version 27 (IBM Corp., Armonk, NY, USA).

Results: While a majority of patients reported acceptable post-stent QOL, many experienced pain and work-related difficulties. Pain was the most common complication, followed by sexual and urinary symptoms. The estimated per-patient healthcare cost of stent management ranged from 5,138 to 5,268 Saudi Riyal (SAR; ≈1,370-1,405 USD), reflecting a notable economic burden.

Conclusion: Although DJ stenting remains effective in relieving obstruction, stent-related morbidity, particularly pain and reduced daily functioning, was significantly associated with poorer quality of life and increased healthcare burden. Optimizing perioperative care, patient education, and timely stent removal may help improve outcomes and reduce the overall system burden.

## Introduction

Double-J (DJ) ureteral stents are widely used to relieve upper urinary tract obstruction and to ensure drainage following urological interventions such as stone management or repair of strictures. Despite their clinical value, these stents frequently cause morbidity, including flank pain, urinary symptoms, and sexual dysfunction, which can significantly affect the quality of life (QOL) of patients. QOL is a multidimensional concept that reflects an individual’s physical, psychological, and social well-being and is commonly used to evaluate the overall impact of medical conditions and treatments on daily functioning. In many cases, these symptoms may exceed what would be expected from obstruction alone, making the indwelling stent itself an important contributor to patient discomfort and impaired daily functioning. This distinction is clinically relevant because it helps contextualize the additional quality-of-life burden experienced by stented patients beyond the underlying urinary tract condition. Previous studies have reported that 80-90% of patients with indwelling stents experience discomfort or reduced daily functioning, underscoring the importance of patient-reported outcomes in assessing stent effectiveness [1,2].

Stent-related symptoms represent not only a clinical challenge but also an economic one, as they often necessitate additional medications, hospital visits, and repeat procedures. Various pharmacological and device-based strategies have been proposed to mitigate these symptoms, though results remain inconsistent and an “ideal” stent design has yet to be achieved [3,4].

In regions such as the Middle East, where kidney stone disease is highly prevalent, the need for ureteral stenting is correspondingly high. Recent analyses indicate that countries in the Gulf region, including Saudi Arabia, report among the highest global incidences of urolithiasis, largely attributed to climatic and dietary factors [5,6]. However, limited local data are available regarding the quality-of-life impact and healthcare costs associated with stent use.

Therefore, this study aimed to evaluate the effects of DJ ureteral stents on stent-related quality of life, which was the primary outcome measured using the Ureteral Stent Symptom Questionnaire (USSQ) [7], and to estimate their economic burden on the healthcare system in Saudi Arabia through a retrospective cross-sectional analysis.

## Materials and methods

This study was conducted as a retrospective cross-sectional survey at Security Forces Hospital (SFH), a tertiary care center in Riyadh, Saudi Arabia, covering cases between January 2021 and January 2024. The research evaluated the impact of DJ ureteral stents on patient outcomes and healthcare system implications. This study was classified as retrospective because all included patients had already undergone DJ stent insertion prior to the initiation of data collection. Clinical and procedural data were retrieved from hospital records, while the telephone survey was used solely to retrospectively assess patient-reported outcomes and quality-of-life measures after their prior stent experience. Telephone interviews were administered by trained research personnel using a standardized approach, with consistent phrasing of questionnaire items and clarification provided only when necessary to ensure participant understanding and accurate response recording. No prospective follow-up or intervention was performed. The telephone interviews were conducted at varying time intervals after stent removal, depending on when each patient became reachable; therefore, a fixed follow-up duration was not applied.

Study population

The study population comprised 250 patients who underwent ureteral DJ stent insertion between January 2021 and January 2024 at the Urology Department of Security Forces Hospital (SFH) in Riyadh. Of these, 135 patients were included in the final analysis, yielding a response rate of 55%. The relatively low response rate was primarily attributed to difficulties in communication, patient refusal to participate, and challenges some patients faced in recalling details of their procedures. Given this 55% response rate, there is potential for selection bias. Characteristics of non-responders were not systematically collected beyond basic demographic information in hospital records; therefore, a formal responder versus non-responder comparison could not be performed.

Inclusion criteria included adults who had undergone ureteral stenting based on clinical evaluation and confirmation by a urologist, individuals with continuous follow-up at SFH, those stented for urolithiasis (stones), individuals who were intellectually intact and Arabic-speaking, and those with complete medical records and non-threaded stents. All participants were required to provide informed consent.

Exclusion criteria included individuals with active urinary tract infections, uncorrectable coagulopathies, missing or incomplete data, those who received stents for oncological indications or in the presence of abnormal urinary tract anatomy (e.g., duplex systems), pregnant patients, and children aged 14 years or younger.

All patients who underwent ureteral DJ stent insertion at SFH between January 2021 and January 2024 and met the inclusion criteria were retrospectively identified from hospital records. No randomization or sampling was applied; instead, a consecutive sampling approach was used, in which all eligible patients were contacted for recruitment. Of the 250 patients identified, 135 consented and completed the survey (response rate: 55%).

As this was a retrospective, single-center study, no formal sample size calculation was performed. The final sample size was determined by the number of eligible patients within the study period who consented and completed the survey. The final sample of 135 participants is comparable to or larger than several previously published studies evaluating ureteral stent-related quality of life using the USSQ. While this limited the total number of cases, the study aimed to capture the entire accessible population rather than rely on a pre-specified sample size.

Survey instrument

Data collection was conducted through telephone interviews using the USSQ, a previously validated multidimensional instrument developed by Joshi et al. to assess stent-related symptoms and health-related quality of life [7]. Permission to use the USSQ was obtained from the original developer. The questionnaire evaluates urinary symptoms, pain, general health, work performance, sexual matters, and additional concerns.

For this study, the USSQ was translated into Arabic using a forward-backward translation process performed by bilingual medical professionals to ensure linguistic and contextual appropriateness for the local population. However, the translated version did not undergo formal psychometric validation (e.g., reliability testing or internal consistency assessment), which represents a methodological limitation of this study.

Telephone interviews were conducted to maximize response rates, include patients unable to attend in-person follow-up, and allow clarification of questionnaire items when necessary, thereby improving data completeness and accuracy.

Data collection

Telephone surveys were conducted retrospectively, collecting patients’ self-reported data regarding symptoms experienced during and after stent placement. Trained research personnel administered the survey, ensuring clarity of questions and accurate recording of responses. To minimize interviewer-related variation, the questionnaire was administered in a standardized manner, with consistent phrasing of items and clarification provided only when necessary to ensure participant understanding. Telephone interviews were conducted between January 2 and March 4, 2025. Because patients had their stents removed at different times prior to the interview period, the interval between stent removal and interview varied among participants, which may introduce recall bias.

Data analysis

Collected data were entered, cleaned, and analyzed using IBM SPSS Statistics, version 27 (IBM Corp., Armonk, NY, USA). Descriptive statistics were utilized to summarize demographic characteristics, symptom prevalence, and QOL assessments. Logistic regression analyses were conducted to identify predictors associated with quality-of-life outcomes. Independent variables entered into the regression model included age, sex, comorbidities, and stent-related factors such as duration of stent placement and laterality. These variables were selected based on clinical relevance and prior literature. Potential confounders (age, sex, comorbidities, and stent duration) were adjusted for in the regression models.

Multicollinearity was assessed using variance inflation factors (VIF), and no significant multicollinearity was detected. Assumptions of logistic regression were verified: independence of observations was maintained, linearity of the logit was confirmed for continuous predictors, and model fit was evaluated using the Hosmer-Lemeshow goodness-of-fit test. Results are presented as adjusted odds ratios (AOR) with corresponding 95% confidence intervals (CI).

Quality of life scoring

QOL was assessed using the USSQ. For interpretability, total USSQ scores were pre-specified before analysis and categorized into three levels for descriptive purposes: high (33-82), average (83-99), and low (100-165). These categories were used as an analytic approach to facilitate the interpretation of symptom burden and QOL impairment. For logistic regression, QOL was dichotomized into low QOL versus high/average QOL to identify factors associated with poorer patient-reported outcomes.

As a secondary exploratory analysis, healthcare costs related to DJ stent management were estimated from a healthcare system perspective.

Healthcare cost estimation

An additional analysis estimated the healthcare cost associated with DJ stent management from a system perspective. Item costs were obtained from the governmental procurement portal (NUPCO) and supplemented with private insurance averages. Components included a hydrophilic sensor tip guide wire (180 Saudi Riyal (SAR)), polytetrafluoroethylene (PTFE) guide wire (50 SAR), and a DJ stent (88 SAR, regardless of size: 4.8 Fr, 5 Fr, or 6 Fr), as well as surgical care (5000 SAR). Inclusive of VAT (15%), the estimated per-patient cost ranged from 5,138 to 5,268 SAR (equivalent to approximately 1,370-1,405 USD).

## Results

The urinary symptoms affecting patients' QOL (Table 1) included a high frequency of daytime urination and nocturia. More than one-third of patients (47, 34.8%) reported urinating more than once every hour during the day, while the majority (117, 86.7%) reported waking up more than once to urinate at night, predominantly twice. Also, the majority of participants experienced urgency (40, 29.6%), but they almost never experienced urinary leakage (66, 48.9%). Most of the patients reported rarely (42, 31.1%) or never (38, 28.1%) seeing blood in their urine, and if they had, it was only in small amounts (66, 48.9%). Additionally, most patients (80, 59.2%) expressed dissatisfaction with the prospect of living the rest of their lives with the stent and the associated symptoms.

**Table 1:** Urinary symptoms Urinary symptoms are presented as frequency (n) and percentage (%). Percentages were calculated based on the total study population (N = 135).

Urinary symptoms		
Daytime urinary frequency	Every 4 hours	6 (4.4%)
	Every 3 hours	15 (11.1%)
	Every two hours	31 (23.0%)
	Every hour	36 (26.7%)
	Less than an hour	47 (34.8%)
Nocturia episodes per night	4 times or more	24 (17.8%)
	3 times	16 (11.9%)
	Twice	39 (28.9%)
	Once	38 (28.1%)
	Never	18 (13.3%)
Urgency (rushing to the bathroom)	Always	40 (29.6%)
	Mostly	29 (21.5%)
	Sometimes	30 (22.2%)
	Rarely	8 (5.9%)
	Never	28 (20.7%)
Urge incontinence (leakage before reaching the toilet)	Always	11 (8.1%)
	Mostly	12 (8.9%)
	Sometimes	28 (20.7%)
	Rarely	18 (13.3%)
	Never	66 (48.9%)
Urgency without voiding (false urge)	Always	3 (2.2%)
	Mostly	9 (6.7%)
	Sometimes	12 (8.9%)
	Rarely	18 (13.3%)
	Never	93 (68.9%)
Sensation of incomplete bladder emptying	Always	22 (16.3%)
	Mostly	18 (13.3%)
	Sometimes	33 (24.4%)
	Rarely	26 (19.3%)
	Never	36 (26.7%)
Dysuria (pain during urination)	Always	36 (26.7%)
	Mostly	29 (21.5%)
	Sometimes	29 (21.5%)
	Rarely	19 (14.1%)
	Never	22 (16.3%)
Hematuria (blood in urine)	Always	20 (14.8%)
	Mostly	13 (9.6%)
	Sometimes	22 (16.3%)
	Rarely	42 (31.1%)
	Never	38 (28.1%)
Severity of hematuria	Large amounts accompanied by blood clots	14 (10.4%)
	Large amount	19 (14.1%)
	Small amount	66 (48.9%)
	None	36 (26.7%)
Overall urinary tract problem severity	Severe	24 (17.8%)
	Fairly bad	41 (30.4%)
	Simple	60 (44.4%)
	None	10 (7.4%)
Perceived long-term impact of stent symptoms	Happy	1 (0.7%)
	Satisfied	22 (16.3%)
	I don’t care	5 (3.7%)
	Dissatisfied	33 (24.4%)
	Unhappy	47 (34.8%)
	Very unhappy	27 (20.0%)

The patients' symptoms of pain (Table 2) indicated that, of the 86 patients who experienced pain, the most common location was the bladder area (28 patients, 32.6%), followed by the upper side (21, 24.4%). Additionally, among the 86 patients who reported stent-related pain, discomfort was most commonly experienced while resting (31, 36.0%) or during simple activities (24, 27.9%), while smaller proportions reported pain during moderate activity (17, 19.8%) or only during serious activities (8, 9.3%). Only six patients (7.0%) reported no pain or discomfort during any activity.

**Table 2:** Pain symptoms Pain-related outcomes are presented as frequency (n) and percentage (%) for categorical variables and as mean ± standard deviation (SD) with median for continuous variables. Percentages for activity-related pain are calculated among patients reporting pain (n = 86).

Statement	Category	N (%)
Presence of stent-related pain/discomfort	Yes	86 (63.7%)
	No	49 (36.3%)
Pain severity score (0–10 scale)	Mean ± SD	6.60 ± 2.26
	Median	7
Pain during activities	I feel pain while resting without making any effort	31 (36.0%)
	I feel pain or discomfort with simple activities	24 (27.9%)
	I feel pain when doing moderate activity, such as walking	17 (19.8%)
	I only feel pain or discomfort when doing serious activities	8 (9.3%)
	I do not feel any pain or discomfort when doing any activity	6 (7.0%)
Sleep disturbance due to stent pain	Always	11 (12.8%)
	Mostly	6 (7.0%)
	Sometimes	14 (16.3%)
	Rarely	14 (16.3%)
	Never	41 (47.7%)
Pain during urination	Always	34 (39.5%)
	Mostly	23 (26.7%)
	Sometimes	13 (15.1%)
	Rarely	7 (8.1%)
	Never	9 (10.5%)
Flank/kidney pain during urination	Always	18 (20.9%)
	Mostly	13 (15.1%)
	Sometimes	15 (17.4%)
	Rarely	10 (11.6%)
	Never	30 (34.9%)
Use of analgesics for stent-related pain	Always	31 (36.0%)
	Mostly	13 (15.1%)
	Sometimes	23 (26.7%)
	Rarely	10 (11.6%)
	Never	9 (10.5%)
Impact of pain on overall quality of life	Always	25 (29.1%)
	Mostly	26 (30.2%)
	Sometimes	19 (22.1%)
	Rarely	9 (10.5%)
	Never	7 (8.1%)

The effects of the stent on work performance (Table 3) revealed that the majority of patients experienced varying levels of difficulty in performing both simple (89, 65.9%) and strenuous activities (120, 88.9%). Most patients (88, 65.2%) were employed, with a significant proportion (82, 60.7%) holding full-time jobs. Meanwhile, 13 patients (9.6%) were not working, with the most common reason being sick leave (8, 61.5%).

**Table 3:** Work performance Work performance variables are presented as frequency (n) and percentage (%) for categorical variables and as mean ± standard deviation (SD) with median for continuous variables. Work-related questions were analyzed among employed participants (n = 88).

Statement	Category	N (%)/value
Difficulty with simple activities (e.g., walking short distances, driving)	Always	16 (11.9%)
	I do not do any activities because of the stent	14 (10.4%)
	Medium difficulty	30 (22.2%)
	Minor difficulty	29 (21.5%)
	No difficulty	46 (34.1%)
Difficulty with strenuous activities (e.g., sports, heavy lifting)	Always	7 (5.2%)
	I do not do any activities because of the stent	102 (75.6%)
	Medium difficulty	8 (5.9%)
	Minor difficulty	3 (2.2%)
	No difficulty	15 (11.1%)
Stress or exhaustion related to the stent	Always	28 (20.7%)
	Mostly	19 (14.1%)
	Sometimes	19 (14.1%)
	Rarely	18 (13.3%)
	Never	51 (37.8%)
Feeling calm and peaceful	Always	45 (33.3%)
	Mostly	37 (27.4%)
	Sometimes	27 (20.0%)
	Rarely	18 (13.3%)
	Never	8 (5.9%)
Enjoyment of social life (e.g., visiting friends/relatives)	Always	55 (40.7%)
	Mostly	21 (15.6%)
	Sometimes	17 (12.6%)
	Rarely	9 (6.7%)
	Never	33 (24.4%)
Need for assistance from family or friends	Always	2 (1.5%)
	Mostly	5 (3.7%)
	Sometimes	15 (11.1%)
	Rarely	13 (9.6%)
	Never	100 (74.1%)
Employment status	Full-time employment	82 (60.7%)
	I am not working and looking for a job	9 (6.7%)
	Not working for other reasons	13 (9.6%)
	Part-time work	6 (4.4%)
	Retired for health reasons	11 (8.1%)
	Retirement for other reasons	11 (8.1%)
	Student	3 (2.2%)
Reason for absence from work (if applicable)	Sick leave	8 (61.5%)
	Legal reasons	3 (23.1%)
	Age	2 (15.4%)
Days bedridden due to stent-related symptoms	Mean ± SD	3 ± 5
	Median	1
Half-days with activity limitation due to stent symptoms	Mean ± SD	4.52 ± 0.238
	Median	0
Frequent breaks or shift changes due to symptoms	Always	14 (15.9%)
	Mostly	18 (20.4%)
	Sometimes	7 (8.0%)
	Rarely	12 (13.6%)
	Never	37 (42.1%)
Change in normal work routine	Always	20 (22.7%)
	Mostly	17 (19.3%)
	Sometimes	8 (9.1%)
	Rarely	7 (8.0%)
	Never	36 (40.9%)
Working same number of hours as usual	Always	41 (46.6%)
	Mostly	12 (13.6%)
	Sometimes	6 (6.8%)
	Rarely	6 (6.8%)
	Never	23 (26.1%)

The patients' general health regarding stent placement (Table 4) showed that nearly half of the patients (66, 48.9%) would be satisfied with the prospect of having a stent placed again if advised by their doctor. Additionally, the overwhelming majority (128, 94.8%) reported that local anesthesia and mild sedation were sufficient to alleviate pain and discomfort during the stent removal procedure.

**Table 4:** General health Post-stent removal outcomes and related experiences are presented as frequency (n) and percentage (%) for categorical variables and as mean ± standard deviation (SD) for continuous variables.

Statement	Category	N (%)/value
Perceived frequency of urinary tract infection (UTI)	Always	9 (6.7%)
	Mostly	28 (20.7%)
	Sometimes	33 (24.4%)
	Rarely	29 (21.5%)
	Never	36 (26.7%)
Antibiotic use due to stent placement	Never	44 (32.6%)
	Three times or more	8 (5.9%)
	Twice	19 (14.1%)
	Once	64 (47.4%)
Hospital visits due to stent-related problems	Never	96 (71.1%)
	Three times or more	11 (8.1%)
	Twice	7 (5.2%)
	Once	21 (15.6%)
Willingness to undergo stent placement again (if advised)	Very happy	2 (1.5%)
	Happy	3 (2.2%)
	Satisfied	66 (48.9%)
	Dissatisfied	16 (11.9%)
	Very dissatisfied	20 (14.8%)
	Unhappy	11 (8.1%)
	Very unhappy	17 (12.6%)
Anxiety regarding purity during prayer due to the stent	Always	16 (11.9%)
	Mostly	13 (9.6%)
	Sometimes	15 (11.1%)
	Rarely	7 (5.2%)
	Never	84 (62.2%)
Difficulty maintaining ablution (wudu) for prolonged periods	Always	26 (19.3%)
	Mostly	20 (14.8%)
	Sometimes	14 (10.4%)
	Rarely	7 (5.2%)
	Never	68 (50.4%)
Response to involuntary leakage during prayer	Complete the prayers and then perform ablution again for the next prayers	6 (4.4%)
	I keep praying and ignore it	6 (4.4%)
	Stop praying and perform ablution again	123 (91.1%)
Delay in performing prayers on time due to the stent	Mostly	6 (4.4%)
	Sometimes	5 (3.7%)
	Rarely	5 (3.7%)
	Never	119 (88.1%)
Need for more frequent ablution than usual	Always	23 (17.0%)
	Mostly	16 (11.9%)
	Sometimes	14 (10.4%)
	Rarely	10 (7.4%)
	Never	72 (53.3%)
Adequacy of local anesthesia and mild sedation during stent removal	I felt severe pain	4 (3.0%)
	I felt some pain	3 (2.2%)
	Yes, it was enough	128 (94.8%)
Pain score during stent removal (0–10 scale)	Mean ± SD	0.64 ± 1.957
Post-removal dysuria	Always	14 (10.4%)
	Mostly	4 (3.0%)
	Sometimes	28 (20.7%)
	Rarely	43 (31.9%)
	Never	46 (34.1%)
Post-removal hematuria frequency	Always	9 (6.7%)
	Mostly	5 (3.9%)
	Sometimes	18 (13.3%)
	Rarely	55 (40.7%)
	Never	48 (35.6%)
Severity of post-removal hematuria	Large amounts accompanied by blood clots	8 (5.9%)
	Large amount	8 (5.9%)
	Small amount	68 (50.4%)
	None	51 (37.8%)
Overall improvement after stent removal	Great improvement	114 (84.4%)
	Slight improvement	14 (10.4%)
	I didn’t feel better	5 (3.7%)
	Symptoms got worse	2 (1.5%)
Post-removal analgesic use	I didn’t need painkillers	88 (65.2%)
	Rarely	18 (13.3%)
	Yes, intermittently	11 (8.1%)
	Yes, daily	18 (13.3%)
Sleep quality after stent removal	I couldn’t sleep normally	2 (1.5%)
	I did not face any difficulty	117 (86.7%)
	I had a slight difficulty	12 (8.9%)
	There was great difficulty in sleeping	4 (3.0%)
Overall rating of the stent removal experience	Bad	5 (3.7%)
	Acceptable	6 (4.4%)
	Good	24 (17.8%)
	Excellent	100 (74.1%)

**Figure 1:**
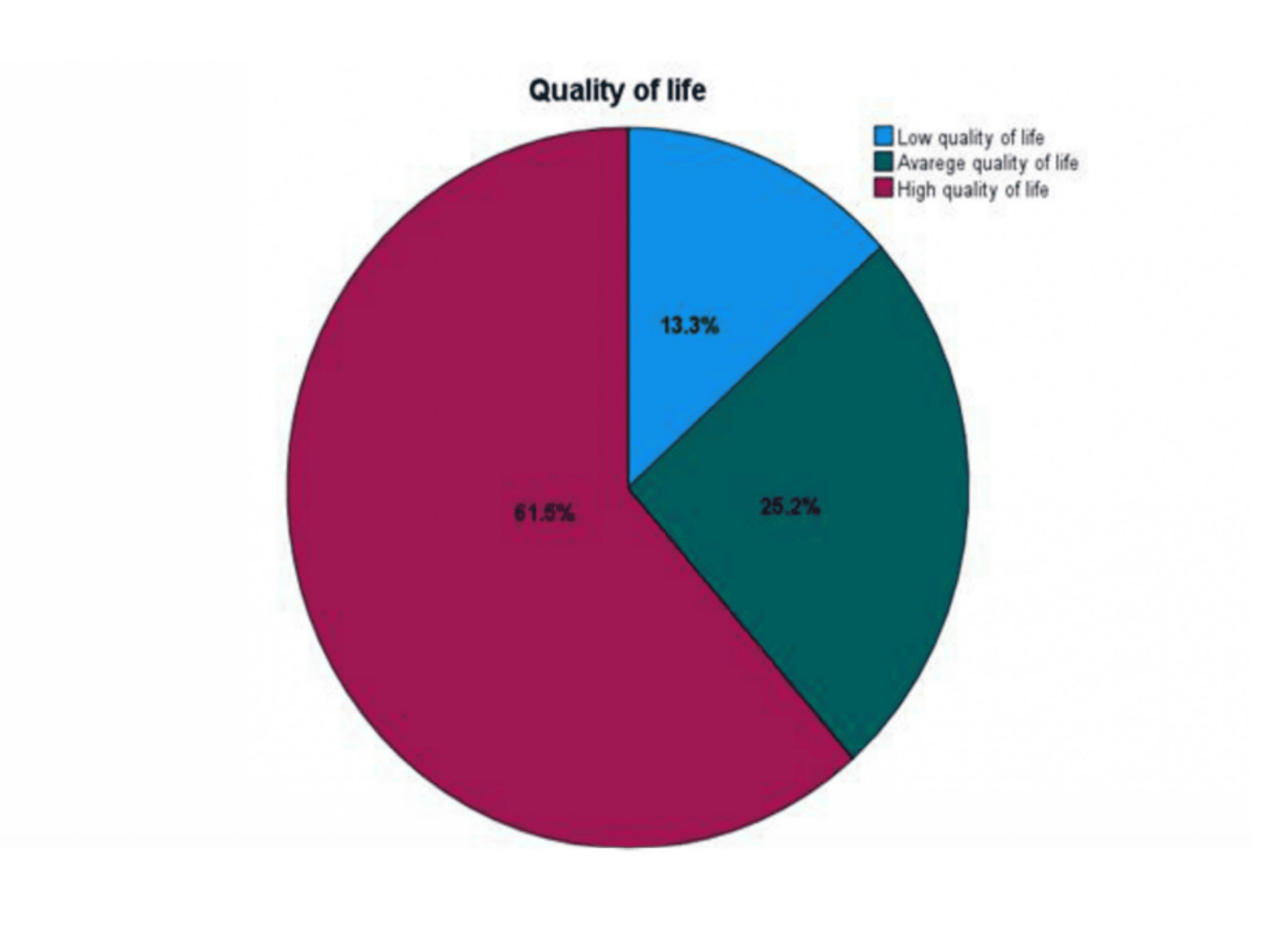
Levels of quality of life (QOL)

QOL (QOL scores) were categorized into three levels: high (33-82), average (83-99), and low (100-165) (Figure 1). Overall, 83 patients (61.5%) reported high QOL, 34 patients (25.2%) had average QOL, and 18 patients (13.3%) experienced low QOL.

The logistic regression analysis (Table 5) showed that pain-related symptoms were most strongly associated with low QOL, with each unit increase in the pain score associated with a 1.71-fold higher likelihood of reporting low QOL (AOR = 1.71; 95% CI: 1.317-2.206; p < 0.001). Sexually related difficulties also contributed substantially, increasing the odds of low QOL by 64% (AOR = 1.64; 95% CI: 1.164-2.315; p = 0.005). Work-related challenges (AOR = 1.43; 95% CI: 1.174-1.732; p < 0.001) and general health symptoms (AOR = 1.42; 95% CI: 1.231-1.647; p < 0.001) were likewise significant, suggesting that the impact of the stent extends beyond physical discomfort to influence daily functioning and overall well-being. Urinary symptoms during stent use (AOR = 1.38; 95% CI: 1.208-1.579; p < 0.001) also played an important role in lowering QOL. In contrast, urinary symptoms after stent removal showed the weakest association with impaired QOL (AOR = 1.32; 95% CI: 1.138-1.579; p < 0.001), indicating that although post-removal symptoms were present, they were less influential compared to symptoms experienced while the stent was in place.

**Table 5:** Factors associated with low quality of life Multivariable logistic regression analysis showing adjusted odds ratios (AOR) with 95% confidence intervals (CI). * Statistical significance was defined as p < 0.05.

Variable	AOR	95% CI	P-value
Pain related symptoms	1.71	1.317 – 2.206	<0.001*
Sexual related difficulties	1.64	1.164 – 2.315	0.005*
Work related difficulties	1.43	1.174 – 1.732	<0.001*
General health symptoms	1.42	1.231 – 1.647	<0.001*
Urinary symptoms	1.38	1.208 – 1.579	<0.001*
Urinary symptoms after stent removal	1.32	1.138 – 1.538	<0.001*

DJ stent management contributes to a notable financial burden on the healthcare system. The estimated per-patient cost includes items such as a hydrophilic sensor tip guide wire (180 SAR), PTFE guide wire (50 SAR), and a DJ stent (88 SAR, regardless of size: 4.8 Fr, 5 Fr, or 6 Fr). In addition, surgical care was estimated at 5000 SAR per patient. The total cost per patient ranged from approximately 5138 to 5268 SAR (Table 6). These figures are based on estimates from the governmental procurement portal (NUPCO). This emphasizes the economic impact of DJ stent usage.

**Table 6:** Estimated local cost of insertion DJ stent Data are presented as costs in SAR and USD. † Only one guide wire is used per procedure (PTFE or hydrophilic sensor tip), producing the cost range. ‡ Estimated total per-patient cost was 5138–5268 SAR (1370–1405 USD). DJ: Double-J; SAR: Saudi Riyal; USD: United States Dollar; VAT: value-added tax; NUPCO: National Unified Procurement Company

Item	Cost per item (SAR)	Cost per item (USD)	Quantity per operation	Total cost per patient (SAR, USD)
Surgical care	5000	1333	1	5000 SAR (1333 USD)
Guide wire (PTFE) †	50	13	1	50 SAR (13 USD)
Guide wire (Hydrophilic sensor tip) †	180	48	1	180 SAR (48 USD)
DJ stent (4.8–6 Fr)	88	23	1	88 SAR (23 USD)
Estimated total per patient ‡	-	-	-	5138–5268 SAR (1370–1405 USD)

## Discussion

Double-J stents play a crucial role in relieving blockage in the urinary tract and maintaining urine flow from the kidney to the bladder [8]. While their value in treating obstruction and supporting postoperative recovery is well established, they are frequently associated with discomfort and complications that negatively affect the patient’s quality of life (QOL) [9]. The present study provides a comprehensive evaluation of these effects by examining patient-reported symptoms, daily functioning, and healthcare burden in a Saudi Arabian tertiary-care setting. Although a majority of patients reported high overall QOL, more than one-third experienced average to low QOL. This highlights the dual nature of stent therapy-clinically effective yet commonly disruptive-a pattern also documented in multiple international studies [10-11].

Pain emerged as the most prominent complication, with 63.7% of patients reporting discomfort and a mean pain score of 6.6/10. This aligns with Kuyumcuoglu et al., who identified pain as the most common and burdensome stent-related symptom [12]. In our study, pain was not only prevalent but also persistent, occurring during rest and simple daily activities and localizing primarily to the bladder and upper abdomen, which are patterns consistent with irritation caused by the stent coils and ureteral distension [13]. The strong association between pain and low QOL (AOR = 1.71) underscores the need for targeted management strategies, including early analgesic therapy, patient education, and consideration of newer stent designs.

In addition to pain, urinary symptoms were also significantly associated with impaired functioning. Patients reported frequency, urgency, nocturia, and discomfort during urination, which are findings comparable to international reports of stent-related lower urinary tract symptoms [14]. Work performance was similarly affected, with many patients reporting difficulty performing even simple tasks and changes in their normal work routine. Sexual difficulties were also reported by a proportion of patients, with some patients reducing or stopping sexual activity due to discomfort or fear of pain. These findings emphasize the multi-dimensional nature of stent-related morbidity, extending beyond physical symptoms to affect psychological, functional, and social aspects of daily life.

A culturally significant dimension emerged in relation to prayer and ablution. A notable proportion of patients expressed anxiety about maintaining ritual purity and reported difficulties performing ablution due to urinary urgency or leakage. Since prayer is obligatory and performed multiple times daily in Saudi Arabia, urinary symptoms may carry a psychological and religious burden beyond their clinical effects. These socio-religious considerations highlight the importance of culturally tailored counseling and reassurance when preparing patients for stent placement.

This study also demonstrated the economic implications of stent management. The estimated per-patient cost of 5138-5268 SAR reflects both procedural expenditures and the downstream utilization of medications and healthcare services. Staubli et al. similarly reported that stent-related morbidity results in significant financial consequences, including increased clinic visits and medication use [15]. Regional findings by Arabi et al. further show that optimization of stent timing can reduce infections and hospitalizations, lowering costs [16]. Our data reinforce the need for strategies such as timely stent removal, structured follow-up, and enhanced symptom control to reduce healthcare burden.

This study has several limitations. Its retrospective cross-sectional design introduces potential recall bias, particularly because the interval between stent removal and telephone interviews was not consistently documented. Selection bias cannot be excluded, as the 55% response rate and lack of detailed data on non-responders prevented a formal responder-non-responder comparison. Although the Arabic version of the USSQ was translated using standard forward-backward methods, it did not undergo formal psychometric validation, which may affect reliability and internal consistency. Additionally, the absence of baseline (pre-stent) QOL data represents an important limitation, as it prevents direct comparison of patient-reported outcomes before and after stent placement and further limits causal interpretation. Other relevant clinical variables, including comorbidities, medication use, stent indication, dwell time, and postoperative complications, were also not comprehensively analyzed, limiting multivariable modeling and assessment of confounding factors. As a single-center study without longitudinal follow-up, generalizability is restricted, and symptom progression over time could not be evaluated. In addition, the economic analysis was limited to a healthcare system perspective and did not account for indirect costs, patient-borne expenses, or broader societal costs, which may underestimate the total economic burden of double-J stent management. Given the retrospective cross-sectional design, the findings should be interpreted as associations rather than causal relationships.

## Conclusions

This study demonstrated that double-J stents, while effective in relieving obstruction, are frequently associated with morbidity and poorer patients’ quality of life. Pain was the factor most strongly associated with poorer tolerance, alongside difficulties with work, sexual function, and cultural considerations such as prayer and ablution. These burdens were associated with greater healthcare utilization and notable financial costs, estimated at 5138 to 5268 SAR (approximately 1370 to 1405 USD) per patient. The findings highlight the importance of patient-centered counseling, structured follow-up, and timely stent management. Future research should focus on multicenter prospective studies, formal validation of the Arabic USSQ, and evaluation of interventions and stent design modifications to improve outcomes and reduce healthcare burden.
